# Additional Remarks upon the Value of Veratrum Viride

**Published:** 1852-02

**Authors:** 


*Additional Remarks upon the value of Veratrum Viride.* By W. C. Norwood, M. D., of Cokesbury, S. C.

We have been endeavoring for some time, to awaken and interest the profession in the powers and properties of Veratrum Viride. We now venture again, and the third time, to call aloud! We have been cautiously, and, as we believe, judiciously, using the above article, alone and in various combinations, for the space of eight years — which ought to allow us to speak with some confidence, and should be a reasonable warrant for what we may assert in the sequel. After various trials and combinations, we unhesitatingly assert that we verily believe we have adopted that form of combination which is *the best.* We gave to the public, in two former numbers of this Journal, (see June No. for 1850, and January No. for 1851,) a portion of our experience, with a statement of what were the powers and properties of the article; and we give this in further addition. Its powers are perhaps more strikingly manifested in the speed and certainty with which it relieves and cures pneumonitis typhoides. Its culminating curative powers stand out in all probability more strikingly in this than in any other disease. We fearlessly assert, that it is as much of a specific in pneumonitis as quinine is in the treatment of intermittent fever, and that it will cut short and break up at the first outset of the attack, in as many cases — due allowance being made for the violence of the attack and the importance of the organs affected — as quinine will, of intermittent. We challenge the world to produce its equal, either singly, or in any combination of remedies. Again: it is the sheet-anchor in typhus and in typhoid fevers. It is the only remedy that has ever been found to arrest the above fever or fevers, and to rob them of the terror and dismay they are known so universally to produce. It not only cures cases beyond the reach of any known remedies, in the last stage of the disease, but it breaks up many cases at the outset, and cuts short others that are fully formed, and in full and perfect progress. Farther: we have found nothing that arrests convulsions in children, accompanied with high febrile symptoms, from one year old and upwards, with any thing approximating such certainty and speed. In hooping cough, it stands unrivaled and alone, as a remedy that may be relied on when accompanied with high febrile excitement. Dr. J. A. Stewart, in writing to us in relation to its powers, states thus: — "I know of no remedy worth mentioning, save yours. Having seen cases of pertussis every day for ten months, and used your remedy every few days, I cannot recommend it to that notice it deserves without being considered as

---

an enthusiast in its use." We hasten on to notice, farther, that it is a powerful and reliable agent in the treatment of typhoid dysentery: that with it we can readily manage that fearful, malignant and mortal disease.

The next class of diseases we shall notice, and briefly illustrate its powers in by a couple of cases, is the certainty and speed — we mean undoubted certainty — with which it cures or relieves the pain and febrile excitement occasioned in mumps, by a metastasis to the testicle.

Case 1st, Mr. A. — Found him with the testicle much swelled and intensely painful; great pain in the head; skin hot and dry; pulse 110; tongue thickly coated. Commenced with eight drops every three hours, the dose to be increased one drop every portion till nausea or vomiting occurred. The third portion excited free emesis. The pulse was reduced to 65 pulsations per minute, the skin became cool and moist, and there was perfect relief of the pain and all unpleasant febrile symptoms were subdued and removed, and by continuing the remedy in small portions, so as not to sicken, there was no return of either pain or febrile symptoms. The symptoms of Mr. S., in the second case, were similar to the first, being free from pain, in the head excepted. He was treated in the same manner precisely. The third or fourth portion vomited freely, on the occurrence of which the pulse was reduced to sixty beats per minute, which before commencing was upwards of 110, with an entire removal of all pain and febrile excitement.

In traumetic lesions we have tested its powers sufficiently to warrant us in asserting that it will control and regulate any arterial excitement produced thereby. We fully tested that fact in the New York emigrant's hospital. Who can calculate its value and importance, by the ease and certainty with which it controls and subdues high arterial excitement after capital operations? How many cases run down and perish from high sanguineous excitement alone, without any other appreciable cause, after well executed operations? We feel confident that in the above we can afford the surgeon a remedy that will quiet his fears and remove his apprehensions in such cases, and that he can control at will inflammation, arterial and general sanguineous excitement, that so often supervene and defeat the successful result of the most skillfully executed operations in surgery.

We feel fully assured, that we can confidently offer to the world the desideratum so long sought and wished for, namely, an agent that will certainly and undoubtedly control and subdue morbid arterial excitement, the great frequency of the contractions of the heart and arteries, so especially belonging to all acute diseases, and the removal of which has been as difficult as its presence was universal in all severely acute diseases. Dr. Bass, writing on the subject, observes, "It seems to act directly upon the heart and arteries, as manifested by a diminution of the force and frequency of the pulse; it relieves irritation, congestion and inflammation — establishes the equilibrium of the circulation — excites free diaphoresis and expectoration, which well adapts it for the treatment of pneumonitis, pneumonia typhoides and asthma — in which diseases I have used it effectually, or in other words, with unparalleled success." Dr. J. Branch, in writing us on the same, states, "I will simply say, I regard it as one of the most important articles of the materia medica. You never made a more just and appropriate remark, than you did when you said, it would say to "the pulse thus fast shalt thou beat and no faster." I have used it in many cases of the severest sort of typhoid fever, with the happiest effect; it will cool the surface, reduce the *frequency* of the pulse, while at the same time it does not diminish its volume or strength. Indeed, I have sometimes thought that the volume and strength of the pulse was increased, in atonic cases, under the use of this article. The following will serve as an illustration of its use and effects: — When called to a case of typhoid fever — with a hot surface, frequent pulse, great restlessness, in a word, with all the symptoms of such a case — if the patient be an adult, I commence with giving him eight drops of the article every two hours, and increase the dose a drop or two at every succeeding dose, until slight nausea is produced, never fearing but that when this effect is produced I shall have a cool surface, an infrequent pulse, and an absence of all febrile excitement. I then continue more or less of the article, until the case is broken up." Dr. J. A. Stewart, in a letter on the same subject, writes thus: "I do not believe any remedy or combination of remedies possesses the same powers in pneumonia or pleuritis as yours — it not only lessens the frequency of the pulse, but exerts a curative influence on the disease, and with regard to its lessening the frequency of the pulse, I unhesitatingly say, without fear of successful controversy, that it will control the pulse in *any* and *every* case where it is morbidly excited. I regard your "remedy" as peculiarly adapted to the treatment of pneumonitis, pleuritis, pneumonia typhoides, pertussis, typhus fever with increased action of the heart and arteries. Mr. Rogers, in whose family you practice, was attacked with typhoid pneumonia about the time you left home, and Drs. Agnew and Traynham attended him, and when all hope of his recovery was lost, his family recollected that some of them had been rescued from an untimely grave by your remedy — urged the physicians to give the "drops." Neither of the physicians having the medicine, they determined to send to me for it; and, with only ʒij of the tincture, both of the physicians assured me they had saved Mr. Rogers, and would not take less than five dollars for the remnant of the two drachms."

We have every confidence that it will cure scarlet fever, also that it will be a valuable remedy in puerperal fever. We are waiting an opportunity to test its powers in the treatment of yellow fever. If we should succeed in curing yellow fever or materially lessening its fearful mortality, who will not hail it the master discovery of the age?

Above we have given a brief outline in addition to what we have heretofore published. We intend shortly to give a full detail of the powers of the article and our entire experience. We challenge the world to discredit the above. We pledge ourselves, and stand ready to demonstrate the powers and effects claimed. We have staked our reputation for veracity and medical skill on the above, and we are perfectly willing to abide the verdict of a liberal and enlightened profession and an intelligent community. Truth is omnipotent. The above was not got up in a day, or a corner, but is the result of years of laborious investigation, and of time and money spent to prove and test the certainty and correctness of our experience, and the conclusions reached, the world can either receive it or reject it.*

[The preparation of Veratrum Viride used by Dr. N. is the saturated tincture of the root. — Ed.] — *Southern Med. and Surg. Journal.*

---

* If a tithe of what is claimed in this article be true, the remedy deserves the attention of practitioners. — Editor Buffalo Journal.

